# Metabolic Adaptation of a C-Terminal Protease A-Deficient *Rhizobium leguminosarum* in Response to Loss of Nutrient Transport

**DOI:** 10.3389/fmicb.2017.02617

**Published:** 2018-01-04

**Authors:** Dong Jun, Zoran Minic, Supriya V. Bhat, Elizabeth M. Vanderlinde, Chris K. Yost, Mohan Babu, Tanya E. S. Dahms

**Affiliations:** ^1^Department of Chemistry and Biochemistry, Research and Innovation Centre, University of Regina, Regina, SK, Canada; ^2^Department of Biology, Research and Innovation Centre, University of Regina, Regina, SK, Canada

**Keywords:** ABC transporters, amino acid metabolism, C-terminal protease, proteomics, *Rhizobium leguminosarum*

## Abstract

Post-translational modification expands the functionality of the proteome beyond genetic encoding, impacting many cellular processes. Cleavage of the carboxyl terminus is one of the many different ways proteins can be modified for functionality. Gel-electrophoresis and mass spectrometric-based techniques were used to identify proteins impacted by deficiency of a C-terminal protease, CtpA, in *Rhizobium leguminosarum* bv. *viciae* 3841. Predicted CtpA substrates from 2D silver stained gels were predominantly outer membrane and transport proteins. Proteins with altered abundance in the wild type and *ctpA* (RL4692) mutant, separated by 2D difference gel electrophoresis, were selected for analysis by mass spectrometry. Of those identified, 9 were the periplasmic solute-binding components of ABC transporters, 5 were amino acid metabolic enzymes, 2 were proteins involved in sulfur metabolism, and 1 each was related to carbon metabolism, protein folding and signal transduction. Alterations to ABC-binding-cassette transporters, nutrient uptake efficiency and to amino acid metabolism indicated an impact on amino acid metabolism and transport for the *ctpA* mutant, which was validated by measured amino acid levels.

## Introduction

Post-translational modification plays a key role in many cellular processes such as the cell cycle (David, 2012), signaling (Mowen and David, 2014), protein–protein interactions (Nogueira-Ferreira et al., 2013) and many others. To date, over 200 types of post-translational modifications have been identified, dramatically increasing the complexity of the proteome (Chandramouli and Qian, 2009). The most common modifications include phosphorylation, acetylation, glycosylation, amidation, hydroxylation, and methylation (Han and Martinage, 1993). Proteolytic post-translational modification is irreversible, ubiquitous and often activates or inactivates proteins by generating shorter protein chains with altered function.

The carboxyl terminal protease A (CtpA) of *R. leguminosarum* bv. *viciae* 3841 is part of a novel group of serine proteases involved in the maturation of other proteins (Gilbert et al., 2007; Rawlings, 2013). The crystal structure of CtpA purified from *Scenedesmus obliquus* shows three domains, including a PDZ domain capable of recognizing short amino acid motifs at the C-termini of target proteins (Liao et al., 2000; Lee and Zheng, 2010). The serine/lysine (Ser/Lys) catalytic dyad catalyzing the hydrolysis reaction of CtpA is distinct from other known serine proteases (Liao et al., 2000; Ekici et al., 2008). Using partially purified Ctp from spinach, Taguchi et al. (1993) showed a preference for P1 residues that are small and uncharged. *Escherichia coli* Tsp will proteolytically process a non-substrate when the WVAAA sequence is added to the C-terminus (Keiler et al., 1996) or when it replaces polar and charged (RSEYE) residues (Parsell et al., 1990). The cleavage site specificity of Ctp is broad, with Ala, Ser, Val, and to a lesser extent, Ile, Leu, Lys, or Arg, preferred at the P1 position (Keiler and Sauer, 1995), and these same residues plus Met, Tyr, or Trp at the P1′ position (Keiler et al., 1995).

CtpA is important for the photosynthetic system of higher plants and algae, helping to rapidly turnover the D1 protein at the reaction center of photosystem II (Bowyer et al., 1992). CtpA in *E. coli* cleaves the penicillin binding protein 3 (PBP3) (Hara et al., 1989, 1991) and in *Borrelia burgdorferi* it processes outer-membrane-associated proteins P13, BB0323, and OspC (Östberg et al., 2004) that form ion channels (Bárcena-Uribarri et al., 2014), help cells persist *in vivo* (Kariu et al., 2013), or assist in host invasion (Tilly et al., 2006), respectively.

To fully understand the cellular functions impacted by a protease, its substrates must be identified, along with the associated processing events that ultimately define its function. C-terminal proteolysis shortens protein chains to produce neo-C-termini, making it difficult to predict the protein’s functional sequence based on the genetic sequence (Tholen et al., 2013). To compound the problem, the widely diverse proteins found in cells have a dynamic range of expression levels, which includes the proteases and their target protein substrates (Vogel and Marcotte, 2012). Techniques developed to determine proteolytic function and substrate identity can be divided into gel electrophoresis- or LC-MS/MS (liquid chromatography-mass spectrometry/mass spectrometry)-based techniques (Otto et al., 2014). One- or two-dimensional (2D) electrophoresis has been successfully used to identify protease substrates (Agard and Wells, 2009) based on shifts from higher to lower molecular weight, reduction in spot intensity, or the appearance or disappearance of protein spots. Substrate spots can be excised and identified by MS, but the results are limited by the complexity of protein mixture and reproducibility (Chandramouli and Qian, 2009). The use of two-dimensional difference gel electrophoresis (2D DIGE) to separate fluorescent dye labeled protein samples allows determination of protein abundance by direct comparison of the dyes used to label treated and control samples (Bredemeyer et al., 2004). A variation of this approach, in which protease substrates of interest migrate off the diagonal on the second post-proteolysis separation, provides some improvement in substrate detection (Shao et al., 2007). In addition, 2D electrophoresis in combination with LC-MS/MS, allows a highly dynamic range in sensitivity, greatly improving throughput and proteome coverage (Chandramouli and Qian, 2009).

Altered substrate processing can impact many cellular processes, including cell envelope integrity. The *R. leguminosarum ctpA* null mutant 3845 has a compromised cell envelope (Gilbert et al., 2007), and is incapable of developing fully mature biofilms, consistent with its altered surface ultrastructure, greater roughness and stronger adhesion to hydrophilic surfaces (Jun et al., 2011). There was no change in the structure of the peptidoglycan peptide bridge for the ctpA mutant, so either PBPs are not substrates of CtpA or there is functional redundancy in the *R. leguminosarum* genome (Jun et al., unpublished). Here we use 2D electrophoresis and MS-based methods to identify proteins impacted by or potential substrates of CtpA and the influence of the *ctpA* mutation on cellular function.

## Materials and Methods

### Materials, Bacterial Strains, and Growth Conditions

All chemicals were purchased from Sigma (Canada) unless otherwise stated. *R. leguminosarum* bv. *viciae* 3841 and the *ctpA* mutant strain 3845 were grown at 30°C in Vincent’s minimal medium (VMM) (Vincent, 1970) or in tryptone-yeast (TY) medium (Beringer, 1974) supplemented as required with 100 μg/mL neomycin and 500 μg/mL streptomycin.

### Two Dimensional (2D) Electrophoresis

Starter cultures of *R. leguminosarum* were used to inoculate TY broth medium cultured to mid-exponential phase (OD_600_ = 0.6), cells harvested (20 min, 8000 × g), the pellets washed (5×, 10 mM Tris HCl, pH 8.5, 10 mM magnesium acetate), and sonicated (10 pulses 8×) in cell lysis buffer (30 mM Tris, pH 8.5, 7 M Urea, 2 M Thiourea, 4% [3-cholamidopropyldimethyl ammonio]-1-propane sulfonate (CHAPS) w/v, 1mM PMSF). Following centrifugation, the supernatant was precipitated and washed (80% acetone) and purified with the ReadyPrep 2D purification kit (Bio-Rad) to eliminate streaks. Concentrations of proteins dissolved in cell lysis buffer were determined by Bradford assay using the Coomassie Plus Reagent (Thermo Scientific) and absorbance at 595 nm (Synergy^TM^ HT Multi-Mode Microplate Reader, BioTek).

Protein was dissolved in 2D rehydration buffer (8 M urea, 2 M thiourea, 4% DTT w/v, 2% CHAPS w/v, 0.1% Bio-lyte 3/10 ampholytes v/v with trace bromophenol blue, 300 μl final volume), loaded onto 17 cm immobilized pH gradient strips with pH ranges of 3–10 and 4–7 (Bio-Rad) covered with mineral oil and separated (14 h, 50 V). Narrow-ranged IPG strips were used for optimal resolution for isoelectric focussing (PROTEAN^®^ i12^TM^ IEF System, Bio-Rad) in rapid mode (30,000 Vh, 10,000 V), reduced from 43,000 Vh to eliminate vertical streaking and improve separation. After the first dimension, strips were incubated (15 min) sequentially in sodium dodecyl sulfate (SDS) equilibration buffer A (6 M urea, 2% SDS w/v, 0.375 M Tris, pH8.8, 20% glycerol w/v, 2% w/v dithiothreitol (DTT)) with slow shaking, followed by the same buffer with 2.5% iodoacetamide (w/v) replacing DTT. The strips were placed on top of the SDS – polyacrylamide gel electrophoresis (PAGE) gels and sealed with 0.5% agarose (w/v) for the second dimension at 4°C using 10–20% gradient acrylamide gels (Jule Biotechnologies) and proteins visualized using silver stain (sensitized in 0.02% Na_2_S_2_O_3_, chilled with 1% AgNO_3_) and developed (2% Na_2_CO_3_, 0.04% formaldehyde in millipore water).

### Difference in Gel Electrophoresis (DiGE)

Proteins from culture were extracted as described above and labeled with dyes according to the manufacturer’s instructions (Lumiprobe) using pH 8.5 for the rehydration buffer for efficient labeling. Protein (50 μg) from wild type and *ctpA* mutant strains were treated with the dyes [400 pmol/μl in dimethylformamide (DMF), Lumiprobe] cyanine 3 and cyanine 5 NHS ester (Cy3 and Cy5), respectively, the protein internal standard treated with Cy2, each incubated (30 min, 4°C, dark), reactions halted with lysine (1 μl, 10 mM), mixed by vortex and incubated (10 min, 4°C, dark). Two samples of each, labeled with different cyanine dyes, and internal standard were mixed and combined with 2× sample buffer [8 M urea, 2% CHAPS, 50 mM DTT, 0.2% Bio-lyte 3/10 ampholytes (v/v), trace bromophenol blue] and dissolved in 2D rehydration buffer (8 M urea, 2 M thiourea, 4% DTT w/v, 2% CHAPS w/v, 0.1% Bio-lyte 3/10 ampholytes v/v and trace of bromophenol blue) to a final volume of 300 μl. Samples were loaded onto 17 cm immobilized pH gradient strips with pH ranges of 3–6, 5–8, and 7–10 (Bio-Rad Laboratories), covered with mineral oil and separated (14 h, 50 V) followed by SDS – PAGE in the second dimension as outlined above, but without staining.

2D gels were imaged with a Typhoon^TM^ Imager and processed using DeCyder Differential Analysis Software v6.5 (Amersham Pharmacia Biotech). Protein spots were detected (differential in-gel analysis), manually checked to exclude artifacts, aligned and analyzed (biological variation analysis). Spot intensities were normalized to the internal standard. For each spot, average abundance with standard deviation of each was compared and statistically analyzed using a student’s *t*-test.

### Protein Identification by LC-MS/MS

Prior to spot-picking, 2D gels were stained with colloidal Coomassie Blue G250 according to the method described by Dyballa and Metzger (2009). Gel plugs were manually excised and washed (3×, 30% acetonitrile in 100 mM NH_4_HCO_3_ pH 8.5), dehydrated (15 min, 100% acetonitrile), dried (15 min, 35°C) in a Savant SpeedVac Concentrator (Thermo Electrition Corporation), trypsin solution (10 μl; 13 ng/μl in 50 mM NH_4_HCO_3_ pH 8.5, 5% acetonitrile) added, incubated on ice (30 min), covered with 50 mM NH_4_HCO_3_ (pH 8.5) containing 5% acetonitrile and digested overnight (37°C). Supernatants from digested samples were desalted (C18-ZipTips, Millipore, Bedford, MA, United States) and used for LC-MS/MS analysis.

Samples were analyzed by nanoLC coupled to the Orbitrap Elite mass spectrometer (MS, Thermo Fisher Scientific) following chromatographic separation of peptides on a Proxeon EASY nLC 1000 (Proxeon, Mississauga, ON, Canada) nano high-performance liquid chromatograph (HPLC). Samples directly injected onto a nano column (C18 column, 10 cm × 75 μm ID, 3 μm, 100 Å) with a eluent (water/acetonitrile/0.1% formic acid, 100 min, 0.30 μl/min) were separated with an acetonitrile gradient: 1–3% (2 min), 3–24% (3–74 min), 24–100% (75–90 min) and a final wash at 100% (91–100 min). Eluted peptides were delivered to the MS using positive electrospray ionization (250°C, 2.1 kV). Full-scan MS spectra (m/z 350–2000) were acquired in the Orbitrap at 60 000 (m/z 400) resolution using automatic gain control settings (1e6 for full FTMS scans and 5e4 for MS/MS scans). Peptides were fragmented with collision-induced dissociation (CID) in the linear ion trap when ion intensity was >1500 counts. The 15 most intense ions were isolated for ion trap CID with charge states ≥ 2 and sequentially isolated for fragmentation using normalized collision energy (35%), activation Q (0.250) and activation (10 ms). Ions selected for MS/MS were dynamically excluded for 30 s. The Orbitrap Elite MS was operated with Thermo XCalibur software.

### Protein and Peptide Identification

RAW MS files were converted into mzXML files for database searching using SEQUEST-PVM v.27 (rev. 9) under standard workflow and a non-redundant rhizobial protein sequence FASTA file from the PATRIC database (Wattam et al., 2014). Search parameters allowed for post-translational modification of methionine by oxidation, and modification of cysteine by carbamidomethylation using precursor mass tolerances of 10 ppm and a fragment mass tolerance 0.6 Da. All peptide matches were filtered by XCorr, mass accuracy (<10 ppm): XCorr >1.5 for +2, +3, and +4 charged precursor ions. A stringent false-discovery rate (FDR) of 1% (or *p* < 0.01) was used to filter candidate peptides.

### RNase Assay

Wild type and *ctpA* mutant strains grown on VMM and TY agar plates for approximately 3–4 days were overlaid with 0.6% agar containing 30 mg/ml type VI Torula yeast RNA (Sigma) and incubated 1 day before the addition of 1 N HCl to precipitate undigested RNA and view the release of periplasmic RNase.

### Amino Acid Analysis

Following centrifugation, the supernatant containing cell-free TY media (1 mL) was collected from lag (8.5 h wt, 24 h *ctpA*), log (19.5 h wt, 34.5 h *ctpA*), early (23 h wt, 47 h *ctpA*) and late stationary (49 h wt, 68 h *ctpA*) phase cultures, frozen (-80°C), lyophilized, dissolved in 200 μL phenylisothiocyanate (PTIC)/water/ethanol/triethylamine solution (1:1:7:1, v/v/v/v), incubated (30 min) and stored frozen (-80°C). Frozen samples were lyophilized, mixed with methanol (200 μL), centrifuged (3 min, 10 000 × *g*) and the supernatants collected for HPLC (Agilent 1100 series) injection. Fourteen amino acids at physiologically relevant concentrations (mM) served as standards. PITC-amino acids (2 μL) were injected onto a reversed phase column (Viva C18, 5 μm, 250 × 4.6 mm (Cat# 9514575, Restek Corporation) with an inline filter and separated by HPLC, equipped with an autosampler and variable wavelength detector, using the method adapted from Dimova (2003). PITC derivatized amino acids were separated in 12 mM sodium phosphate buffer pH 5.5 (A) and methanol (B) at a flow rate of 0.5 mL/min by gradient elution: 65% A from 0 to 5 min; 65–40% A from 5 to 30 min, 40–5% A from 30 to 35 min, 5–90% A from 35 to 40 min, and 90–65% from 40 to 47 min. The relative amount of each PITC derivatized amino acid detected at 254 nm was calculated from peak area.

## Results

### 2D Electrophoresis

Approximately 350 spots were visualized on 2D gel images using a pI range of 3–10 in the first dimension, for which 20 were unique to the wild type and 6 unique to the *ctpA* mutant (Supplementary Figure S1). 2D separation with a pI range of 4–7 gave rise to 550 spots, with 16 unique to wild type and 18 unique to the *ctpA* mutant (Supplementary Figure S2 and Supplementary Table S1), indicating potential CtpA substrates or those indirectly influenced by the *ctpA* mutation. Based on electrophoretic patterns, proteins could be assigned to either potential CtpA substrates, or proteins that have higher or lower abundance in the *ctpA* mutant, and each was identified using the published genome of *R. leguminosarum* (Young et al., 2006).

### Predicting CtpA Substrates in *Rhizobium leguminosarum* bv. *viciae* 3841

Based on the processing sites studied *in vitro* (Keiler et al., 1995, 1996) and the published genome of *R. leguminosarum* (Young et al., 2006), the C-termini of putative cell envelope proteins were examined to identify amino acid residues preferred by C-terminal proteases in the P1, P2, and P3 positions for proteins having differential expression in the wild type and mutant. Proteins with appropriate pI and mass values for the wild type (CtpA processing) and mutant (no processing) from all the silver stained 2D gels having predicted processing sites are shown in Supplementary Table S2.

### Difference in Gel Electrophoresis (DiGE)

**FIGURE 1:**
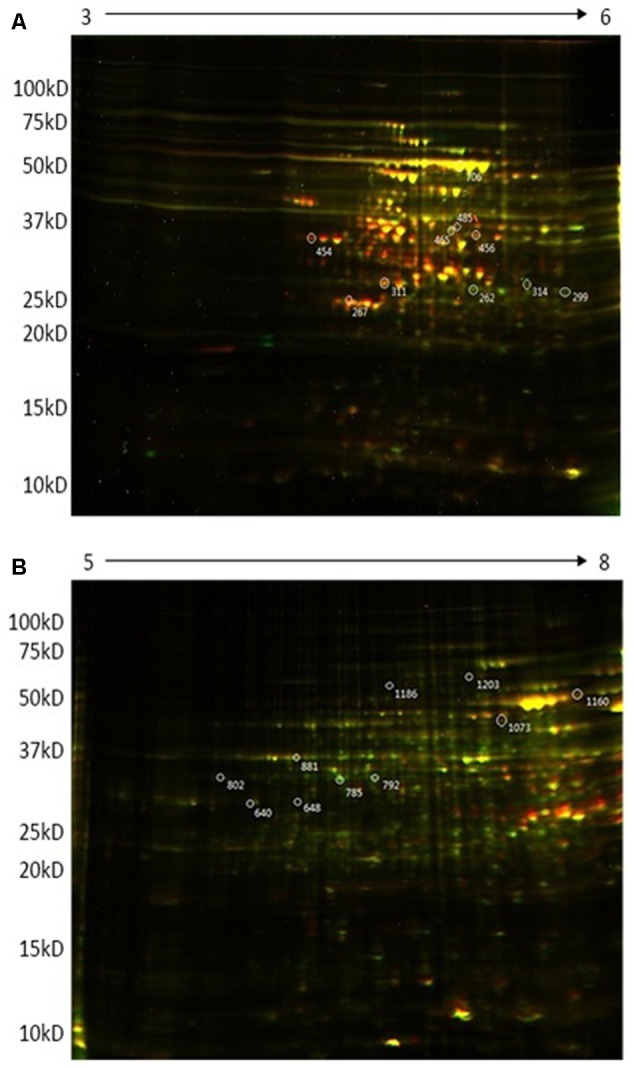
Representative 2D-DIGE expression maps of *Rhizobium leguminosarum* bv. *viciae* 3841 (wt) and 3845 (*ctpA* mutant) labeled with Cy3 (green) and Cy5 (red), respectively. First dimension in **(A)** is pH 3-6 and in **(B)** is pH 5–8.

The more sensitive 2D DiGE electrophoresis showed that the majority of proteins had pIs between 4 and 8. **Figure 1** shows representative DiGE images of four biological replicates for the wild type and *ctpA* mutant of *R. leguminosarum*. Approximately 1200 proteins could be visualized from 2D gels in the pI range 5–8, of which 651 appeared in all four replicates and 301 spots had significantly increased or decreased intensity in gels of the *ctpA* mutant strain. More than 800 proteins were detected using a pI range of 3 to 6, of which 332 protein spots appeared in all four replicates and 170 had higher or lower intensity in the *ctpA* mutant strain. In the pI range of 7–10 (data not shown), 500 proteins were detected in the 2D gels of which 142 protein spots appeared in all four replicates and 3 spots had significantly higher or lower intensity in the *ctpA* mutant strain. Twenty spots, showing significantly increased or reduced abundance in the *ctpA* mutant strain (*p* < 0.001), were selected for further analysis (**Table 1**).

**Table 1:** Major protein components of the *ctpA* mutant from selected 2D-DIGE spots identified by mass spectrometry^a^.

pI range/spot #^b^	Ratio^c^	Gene/protein	Geninfo identifier #^d^	Gene code^e^	# Peptides	Function
5–8/648	1.75	cysD	VBIRhiLeg32091_2444	RL1261	30	Sulfur assimilation
5–8/785, 792	2.06, 2.13	aroG^f^	VBIRhiLeg32091_3937	RL2686	33, 31	Phe, Tyr, and Trp *biosynthesis*
5–8/881	–1.21	aldA	VBIRhiLeg32091_3196	RL1966	17	Ala biosynthesis
5–8/1186	1.48	typA	VBIRhiLeg32091_5846	RL4506	51	Signal transduction
5–8/1203	–1.85	Periplasmic peptide-binding component	VBIRhiLeg32091_7097	pRL110243	52	Solute binding component of ABC transporter
5–8/1073	–1.82	Putative dipeptide/oligopeptide solute-binding component	VBIRhiLeg32091_5914	RL4575	33	Solute binding component of ABC transporter
5–8/640	1.64	Fructose-bisphosphate aldolase class I	VBIRhiLeg32091_5326	RL4012	32	Carbon metabolism
5–8/802	1.33	adhI	VBIRhiLeg32091_0524	pRL120524	32	Glutathione metabolism
3–6/314	2.18	Tricarboxylate transport protein TctABC	VBIRhiLeg32091_5210	RL3891	21	Transport of three carbon sugars
3–6/454	–2.81	Sugar ABC transporter, periplasmic sugar-binding protein	VBIRhiLeg32091_4922	RL3617	22	Transport of sugars
3–6/456, 465	–2.94, -2.82	Putative periplasmic substrate-binding ferrisiderophore receptor^f^	VBIRhiLeg32091_3966	RL2713	28, 27	Solute binding component of ABC transporter
3–6/267	–3.77	Amino acid ABC transporter, periplasmic amino acid-binding protein	VBIRhiLeg32091_4007	RL2753	27	Solute binding component of ABC transport system
3–6/299	1.62	ilvE	VBIRhiLeg32091_2512	RL1326	24	Ile, Leu, and Val biosynthesis
3–6/311	–3.14	Predicted erythritol ABC transporter 2, substrate-binding component	VBIRhiLeg32091_0201	pRL120200	13	Solute binding component of ABC transporter
3–6/262	1.95	Fumarylpyruvate hydrolase	VBIRhiLeg32091_4442	RL3169	14	Tyrosine metabolism
3–6/706	–1.57	groEL^f^	VBIRhiLeg32091_2042	RL0883	46	Protein folding
3–6/485	–2.79	Leu, Ile, Val, Thr, and Ala binding protein	VBIRhiLeg32091_7251	pRL110400	24	Solute binding component of ABC transport system

*^a^Significant difference (*t*-test) in spot intensity between wild type and *ctpA* mutant is for *p* ≤ 0.001. ^b^Protein probability is 99.58%. ^c^Ratio of *ctpA* mutant vs. wild type average spot intensities for identified proteins separated by DIGE. ^d^The sequence accession number of identified proteins from Pathosystems Resource Integration Center (PATRIC). ^e^Gene code is according to the Sanger Centre annotation. ^f^Identified from two different DIGE spots.*

### LC-MS/MS of Proteins with Altered Abundance in the *ctpA* Mutant

**FIGURE 2:**
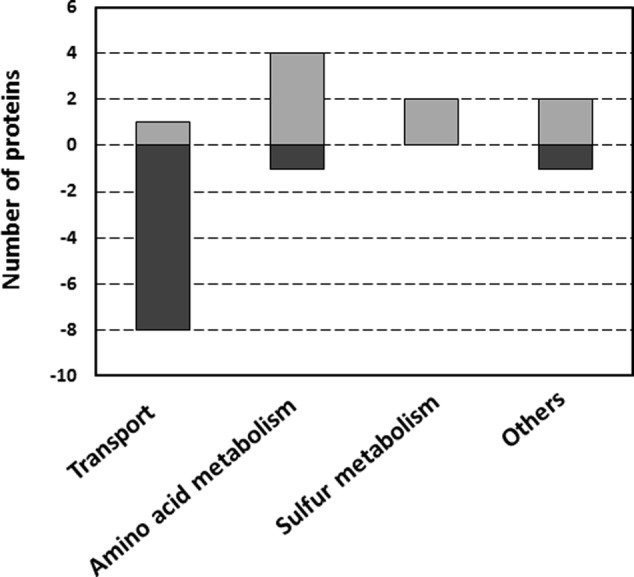
Functional classification of proteins with differential abundance in wild type and *ctpA* mutant *R. leguminosarum*, according to their biological function. The category “Others” includes one protein in each of the following categories: energy metabolism, protein folding and signal transduction. Assignments were made according to the Clusters of Orthologous Groups of proteins database.

Proteins from the selected 20 spots were further identified by LC-MS/MS and those constituting the majority of each are listed in **Table 1**. Some proteins could be linked to certain metabolic pathways using the KEGG database (Kanehisa and Goto, 2000; Kanehisa et al., 2016, 2017). The Clusters of Orthologous Groups of proteins database was used to classify selected proteins of *R. leguminosarum* bv. *viciae* 3841 into six categories according to their function (**Figure 2**).

### RNase Release Assay

The RNase release assay was used to test if the reduction of transporter proteins in the *ctpA* mutant cell envelope is the result of a destabilized outer membrane. Zones of clearing, indicating leakage of periplasmic RNase I into the agar, were observed surrounding the *ctpA* mutant colonies, but not wild type (data not shown).

### Amino Acid Analysis

**FIGURE 3:**
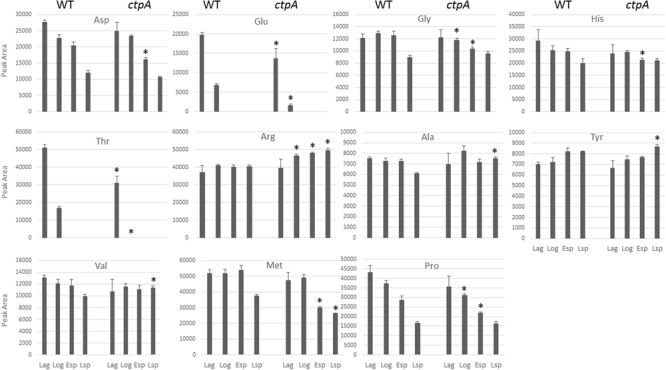
Histograms of peak areas for 11 amino acids from culture at lag, log, early and late stationary phases for the wild type (WT) and *ctpA* mutant. Columns with no bars represent signal not detected. Asterisks represent statistically significant differences relative to wild-type (*p* < 0.05) from a two-tailed student’s *t*-test.

To determine whether amino acid profiles were altered in the *ctpA* mutant, amino acid consumption was measured in lag, log, early and late stationary growth phases (esp and lsp, respectively) by HPLC. Fourteen amino acid standards relevant to this study were chromatographically separated, but high levels of Ile, Leu, and Trp, even in dilute samples (data not shown), precluded adequate resolution and further analysis. As expected, samples without PITC treatment lacked signal at 254 nm, serving as a negative control (Supplementary Figure S3). The relative amounts of PITC-amino acids obtained during the four growth phases for the wild-type and *ctpA* mutant are shown in **Figure 3**. In the mutant, there was a significant (*p* < 0.05) accumulation of Ala, Arg, Val, and Tyr during the late stationary phase, with Arg also accumulating at log and early stationary phase. There was a significant (*p* < 0.05) reduction in Glu and Thr during lag and log phases, Gly and Pro at log and early stationary phase, Asp and His during early stationary phase, and Met at early and late stationary phases in the mutant compared to wild type.

## Discussion

In microbes, proteases have been linked to protein turnover, sporulation, conidial discharge, germination, nutrition and regulation of gene expression (Rao et al., 1998). Proteases are all capable of hydrolyzing the amide bond of peptide and protein substrates despite their varied mechanisms. Proteins expressed as precursors with a cleavable carboxyl-terminal extension (Bhargava and Spremulli, 2005) can be cleaved by CtpA during post-translational modification (Hara et al., 1991). The *ctpA* gene was identified in the genome of *R. leguminosarum* bv. *viciae* 3841 (Gilbert et al., 2007), with the CtpA protein sequence analysis showing highest similarity between members of the *Rhizobiaceae* order, such as *Sinorhizobium meliloti, Agrobacterium tumefaciens* (85% identity), and *Mesorhizobium loti* (75% identity). The *R. leguminosarum* CtpA has sequences homologous to the peptide binding motif and a catalytic dyad of a Ser protease, but its substrates are unknown. Searching for CtpA substrates is challenging but crucial for identifying the role of CtpA in the biological process of *R. leguminosarum* bv. *viciae* 3841, and understanding the downstream effect of CtpA in cellular physiology.

### Proteins Impacted by CtpA Deficiency

Carboxyl terminal protease is hypothesized to be transported into the periplasmic space through the inner membrane following cytoplasmic biosynthesis. Hara et al. (1991) and then Silber et al. (1992) demonstrated the localization of Prc in the cytoplasmic membrane and periplasm of *E. coli*. More recently, however, Hoge et al. (2011) detected only periplasmic CtpA in *Pseudomonas aeruginosa* which was not observable without the introduction of an expression vector harboring the *ctpA* gene, speculating that localization to the cytosol and inner membrane was a consequence of artificial CtpA overexpression.

Carboxyl terminal protease is not a highly specific protease, likely acting on a number of substrates, making it more difficult to identify its exact repertoire. Based on 2D electrophoretic patterns, proteins could be assigned to either potential CtpA substrates or proteins with higher or lower abundance in the *ctpA* mutant (Supplementary Table S2). While these proteins are potential targets of CtpA, actual processing is expected to be limited to those protein precursors having non-polar carboxyl termini. The majority of the proteins predicted in this manner were putative outer membrane proteins and transporter components. A detached outer membrane of the *ctpA* mutant, viewed by TEM (Jun et al., unpublished) and confirmed by the RNase assay, is consistent with previous data (Gilbert et al., 2007) and implies a loss of lipoproteins. Such proteins are widely distributed in Gram-negative bacteria and act as structural proteins to affix the outer or inner membrane to the peptidoglycan layer (Cascales et al., 2002). Indeed, the lipoproteins OspC and BB0323 are processed at the C-termini by CtpA in *B. burgdorferi* (Östberg et al., 2004; Kumru et al., 2011). The C-terminus of integral outer membrane porin P13 and BBA01 are also cleaved by CtpA in *B. burgdorferi* (Noppa et al., 2001; Pinne et al., 2006). So lipoproteins and porins are suspected targets for CtpA in *R. leguminosarum* bv. *viciae* 3841, but were not identified in this study, possibly since such a small proportion of proteins was characterized.

**FIGURE 4:**
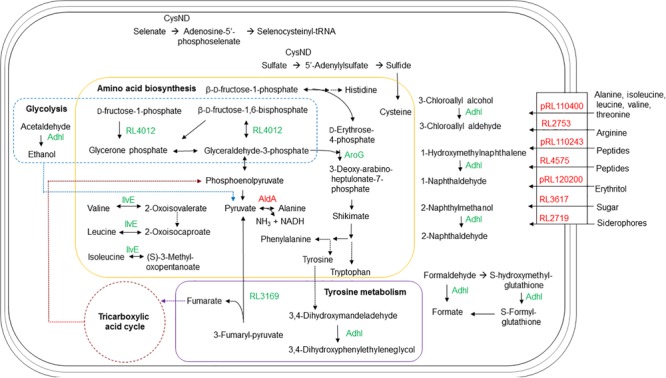
Schematic representation of the metabolic pathways and transport in *R. leguminosarum* bv. *viciae* affected by the *ctpA* mutation. In green are proteins with more abundance in the *ctpA* mutant and in red proteins have lesser abundance in the *ctpA* mutant. Solid arrows indicate a single step, while dashed arrows indicate multiple steps.

2D separation conditions were optimized using silver stain which is incompatible with mass spectrometry, so proteins were isolated by DIGE using optimal separation conditions with small pI ranges for identification by LC-MS/MS (**Table 1**). Identified proteins were distinct from those predicted (Supplementary Table S2), likely based on different pI ranges and the analysis of a relatively small number of spots, but both revealed a number of transport-related proteins. The majority of proteins identified by MS were related to transport and amino acid metabolism (**Figures 2, 4**), supported by validation studies showing altered levels of amino acids from the culture media of the wild type and mutant at lag, log, early and late stationary phases (**Figure 3**).

#### Transport

The putative solute-binding component of an ABC transporter encoded by RL3617 shares 98% similarity with its homolog ChvE in *A. tumefaciens*, a multiple sugar-binding periplasmic receptor (Wood et al., 2001) of the sugar ABC transporter (Kemner et al., 1997). The *chvE* mutant is slower growing, like the *ctpA* mutant (Gilbert et al., 2007), with a delayed chemotactic response to sugars (Shimoda et al., 1993). EryG encoded by pRL120200 is a periplasmic-binding protein for the erythritol ABC transporter (Yost et al., 2006) and its reduced abundance could impact the net transport of erythritol.

The gene products of RL4575 and pRL110243 are predicted to participate in nickel/peptide transport across the *R. leguminosarum* cell envelope. pRL110243 is the homolog of OppB in *E. coli* and *Salmonella typhimurium*, a hydrophobic integral membrane protein responsible for the transport of peptides across the cytoplasmic membrane (Pearce et al., 1992). RL4575 encodes a protein predicted as a putative solute-binding component of an ABC transporter containing a nickel/dipeptide/oligopeptide binding domain. Peptide uptake can play a major role in the nutrition for the organism, and a reduced abundance of these transporters may explain slower growth for the *ctpA* mutant (Gilbert et al., 2007).

The gene pRL110400 is predicted to encode the Ala-, Ile-, Leu-, Val-, and Thr-binding component of an ABC transporter in *R. leguminosarum*. The lower abundance of the pRL110400 gene product in the *ctpA* mutant is validated by the accumulation of Ala and Val at late stationary phase in the mutant’s growth media, but with no accumulation of Thr as might be expected (**Figure 3**). The periplasmic amino acid-binding protein encoded by RL2753 shares identity with two amino acid-binding proteins in *E. coli*, ArtJ (39%) and HisJ (41%), the former being the periplasmic binding component of the L-Arg ABC transport system (Wissenbach et al., 1995). The lower abundance of RL2753 is expected to impair amino acid uptake in the *ctpA* mutant, consistent with accumulation of Arg in the culture media of the *ctpA* mutant (**Figure 3**).

Many of the identified proteins are functionally connected (Supplementary Figure S4), with many involved in the ABC-binding-cassette transport system. Bacterial ABC transporters are involved in many biological processes, including multidrug resistance, protein secretion, quorum sensing, and in this case nutrient uptake (Higgins, 2001; Taga et al., 2001; Chang, 2003; Holland et al., 2005). In Gram-negative bacteria, ABC transporters consist of at least a periplasmic binding protein which binds solutes, a membrane-bound transport protein which interacts with the periplasmic protein and an ATP-binding protein which provides the energy required for transport (Higgins, 2001). In all cases, it was the periplasmic solute binding protein of ABC transporters that was impacted in the *ctpA* mutant (**Table 1**). A low abundance of periplasmic binding proteins in the *ctpA* mutant would affect solute uptake efficiency and response to chemotactic stimuli, further explaining its slow growth rate (Gilbert et al., 2007). The *ctpA* mutation broadly impacts the ABC transport system, which we attribute to a compromised outer membrane (Jun et al., unpublished).

#### Amino Acid Metabolism

Other than RL1966 (**Table 1** and **Figure 2**), proteins involved in amino acid metabolism are found in higher abundance in the mutant, possibly to compensate for reduced nutrient uptake by impaired transport. Alanine dehydrogenase (AldA) encoded by RL1966 is the principle enzyme of *de novo* alanine biosynthesis, catalyzing the reversible conversion of pyruvate, ammonium and NADH to Ala (Lodwig et al., 2004). The lower abundance of AldA in the *ctpA* mutant indicates either less pyruvate or Ala in the cell, consistent with Ala accumulation at late stationary phase in the media (**Figure 3**). IlvE encoded by RL1326 is a branched-chain amino acid aminotransferase responsible for the last step of Ile, Leu, and Val biosynthesis, along with the first step in their degradation (Kanehisa and Goto, 2000; Kanehisa et al., 2016, 2017), in accordance with altered Val levels. The gene *aroG* (RL2686) encodes the feedback regulated enzyme 3-deoxy-D-arabino-heptulosonate synthase (DAHP), part of the shikimate pathway that catalyzes the first step in the biosynthesis of Tyr, Phe, and Trp. Expression of AroG in *Solanum lycopersicum* and *Arabidopsis* plants increases levels of shikimate pathway metabolites, Phe, Tyr, and Trp, along with altered levels of Asn, Gln, Gly, Ile, *N*-acetyl-Glu and Thr (Tzin et al., 2012, 2013), consistent with altered amino acids levels in the *ctpA* mutant (**Figure 3**). RL3169 encoding a predicted protein shares 40% identity with a putative fumarylacetoacetate hydrolase YcgM in *E. coli*, which is involved in tyrosine degradation (Kanehisa and Goto, 2000; Kanehisa et al., 2016, 2017). The higher abundance of Tyr in the mutant at late stationary phase is consistent with the slightly greater increase in RL2686 than RL3169 in the mutant (**Table 1**).

#### Other Proteins

CysD, encoded by RL1261, corresponds to the putative sulfate adenylyltransferase subunit 2 which helps assimilate sulfur. Sulfur is an essential element incorporated into many molecules including the amino acids Cys and Met, as evidenced by reduced Met levels in the stationary phases (**Figure 3**). Putative alcohol dehydrogenase AdhI encoded by pRL120524 shares 58% identity with *S*-(hydroxymethyl) glutathione dehydrogenase frmA in *E. coli* (Gutheil et al., 1992). AdhI is involved in multiple metabolic pathways, including carbon metabolism, catabolism of aromatic compounds, fatty acids, and sugars (glycolysis), methane metabolism, tyrosine metabolism, chloroalkane and chloroalkene degradation (Kanehisa and Goto, 2000; Kanehisa et al., 2016, 2017). RL4012 encodes fructose-bisphosphate aldolase, involved in several reactions of carbon metabolism, glycolysis, the pentose phosphate pathway, amino acid biosynthesis, methane metabolism, fructose and mannose metabolism. Higher levels of pRL120524 and RL4012 may compensate for reduced carbon and amino acid transport.

GroEL encoded by RL0883, detected in two gel spots (**Table 1**), is responsible for proper protein folding, is induced under stress conditions (Goulhen et al., 1998; Kusmierczyk and Martin, 2000; Klančnik et al., 2006) and plays an important role in the export of certain proteins (Kusukawa et al., 1989). Unlike *E. coli, R. leguminosarum* strain A3 has three genes encoding GroEL homologs (Rodríguez-Quiñones et al., 2005). Interestingly, GroEL is found to be down regulated in *Bradyrhizobium japonicum* under acidic conditions (Puranamaneewiwat et al., 2006), consistent with the *ctpA* mutant (**Table 1**) and suggesting it likely plays distinct roles in rhizobia.

TypA (tyrosine phosphorylated protein A) encoded by RL4506, a predicted protein in *R. leguminosarum* bv. *viciae* 3841, shares 56% identity with the GTP-binding protein TypA/BipA in *E. coli* K12. Disruption of *E. coli typA* alters protein expression and modification during exponential growth and carbon starvation (Freestone et al., 1998). TypA is involved in temperature-dependent regulation of *E. coli* cell surface polysaccharides (Rowe et al., 2000) and the survival of *S. meliloti* 1021 under stressful conditions (Kiss et al., 2004). Higher levels in the mutant may reflect carbon deficiency as a result of reduced carbon transport.

In summary, we show an impact to the ABC-binding-cassette transport system in the *ctpA* mutant and thus nutrient uptake efficiency that is consistent with its slow growth rate and a compromised outer membrane. The mutant appears to adapt by increasing a number of metabolic enzymes that would be capable of compensating inadequate nutrient transport. The predicted impact on amino acid metabolism and transport was validated by altered amino acid levels for the *ctpA* mutant.

## Author Contributions

DJ designed and completed most of the experiments, with some assistance from EV and CY, analyzed the data and prepared a first draft of the manuscript. ZM and SB completed the amino acid analysis. MB oversaw the proteomics data and provided data input into data interpretation. TD helped design all experiments, edited and polished the manuscript with editorial input from EV, SB, ZM, CY, and MB.

## Conflict of Interest Statement

The authors declare that the research was conducted in the absence of any commercial or financial relationships that could be construed as a potential conflict of interest.
