# Supplementary material for: Metabolic Adaptation of a C-Terminal Protease A-Deficient Rhizobium leguminosarum in Response to Loss of Nutrient Transport
Source: Front Microbiol. 2018 Jan 4;8:2617. doi: 10.3389/fmicb.2017.02617 (PMC5758756; doi:10.3389/fmicb.2017.02617)
Supplement: Supplementary file 1 [file Image_1.pdf]

Supplementary Data

Metabolic adaptation of a C-terminal protease A-deficient *Rhizobium leguminosarum* in response to loss of nutrient transport

Dong Jun[a], Zoran Minic[a], Supriya V. Bhat[a], Elizabeth M. Vanderlinde[b], Christopher K. Yost[b], Mohan Babu[a] and Tanya E. S. Dahms[a,*]

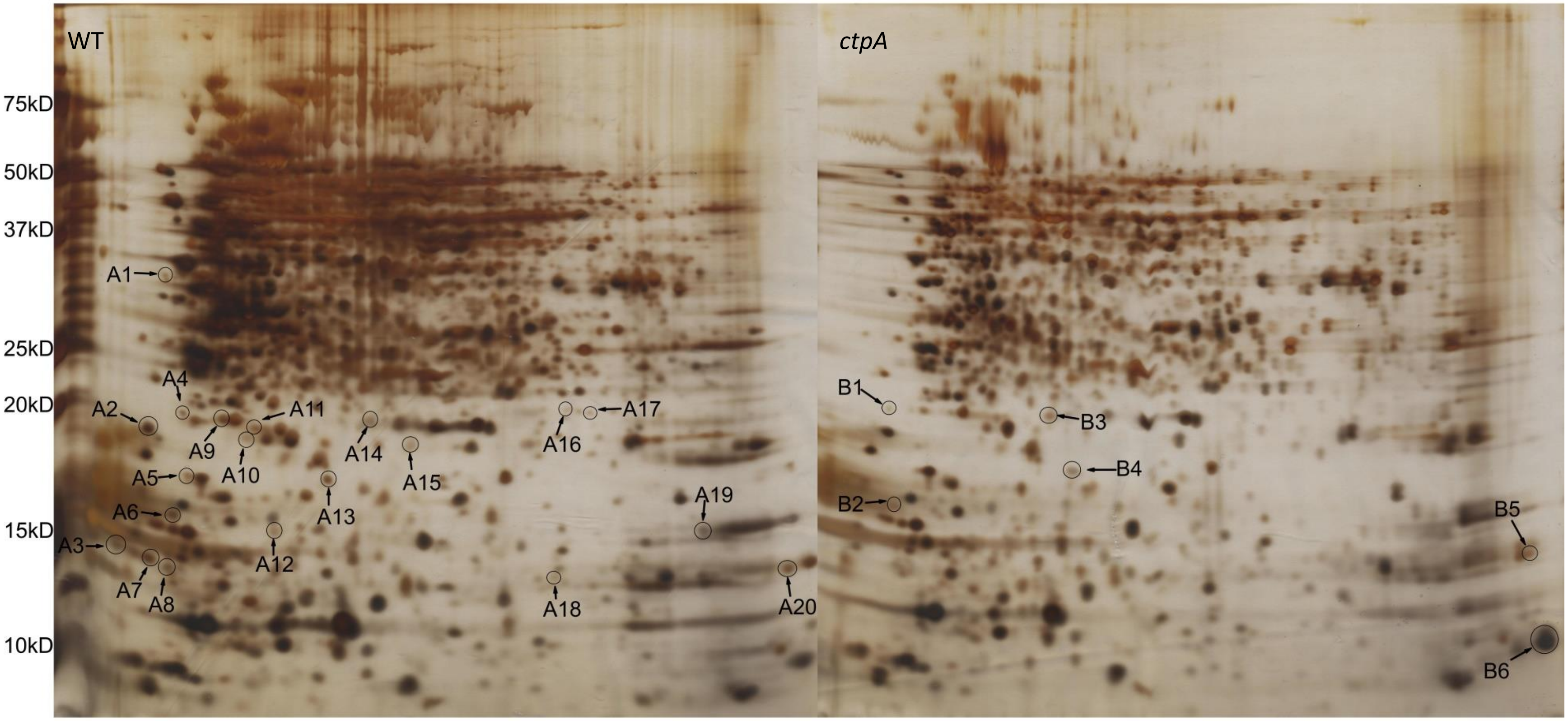

Figure S1. Silver stained whole cell 2D electrophoresis protein gel profiles of *Rhizobium leguminosarum* bv. *viciae* 3841 (WT, left) and the *ctpA* 3845 mutant strain (*ctpA*, right). Proteins were initially focused on 17 cm IPG strips (pH 3-10) for a total of 30,000 Vh, followed by SDS PAGE electrophoresis on 12% acrylamide gels. Spots A1-20 are unique to WT and B1-6 are unique to the *ctpA* mutant.

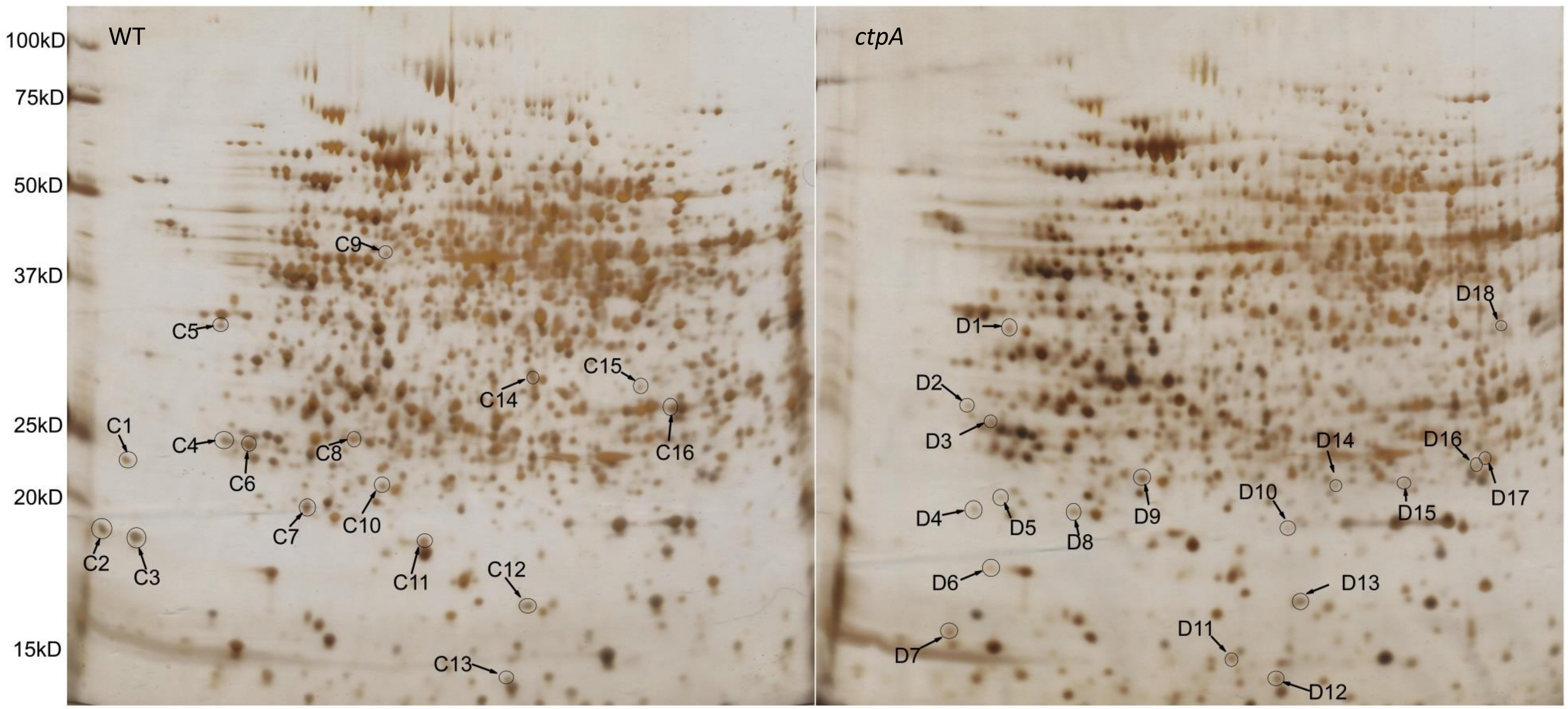

Figure S2. Whole cell 2DE protein gel profiles of *Rhizobium leguminosarum* bv. *viciae* 3841 (WT, left) and *ctpA* 3845 mutant strain (*ctpA*, right). The proteins were focused on 17 cm IPG strips (pH 4-7) for a total of 30,000 Vh followed by SDS PAGE electrophoresis on 10% acrylamide gels. C1-16 are unique to WT and D1-18 are unique to the *ctpA* mutant.

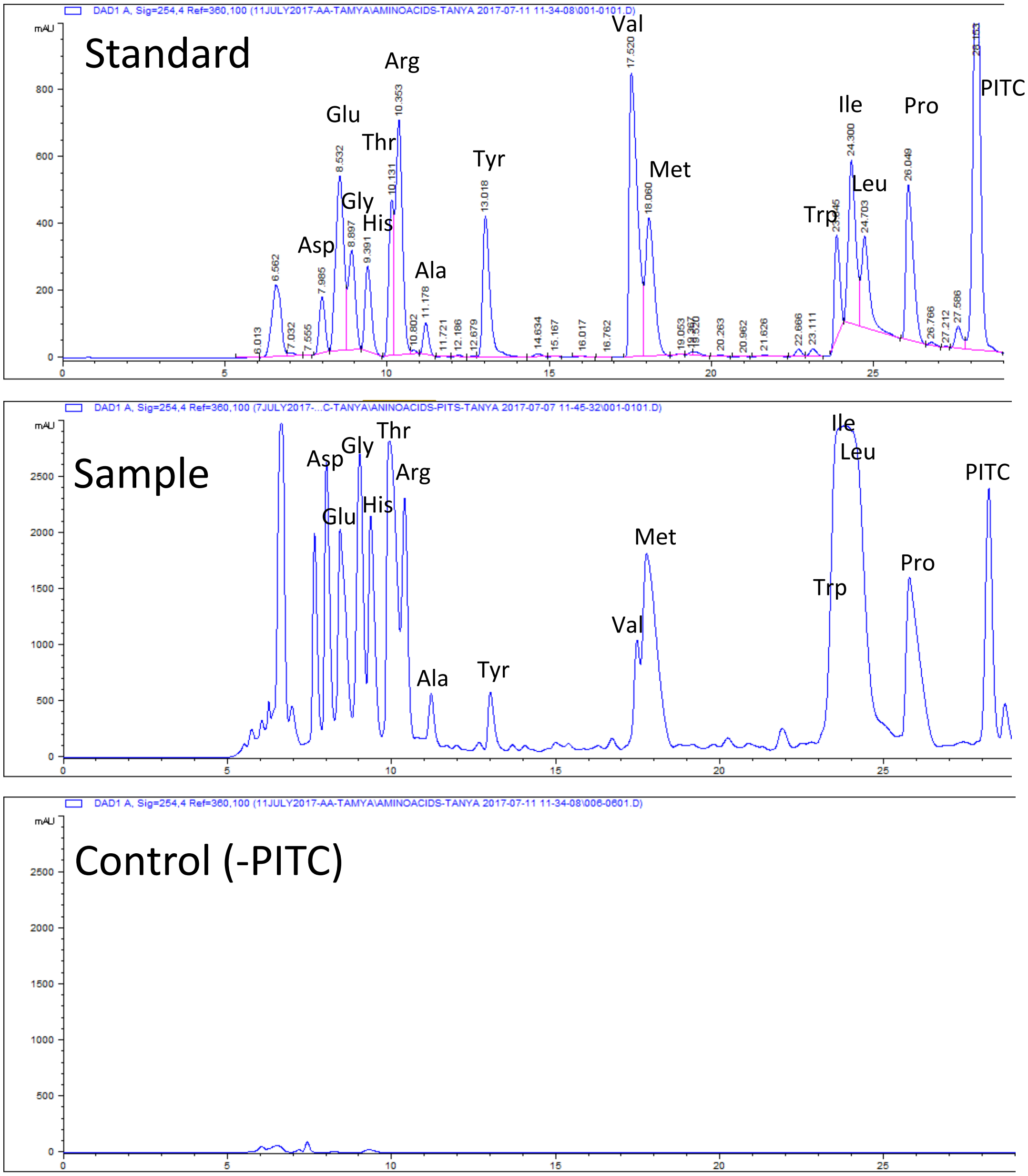

Figure S3. Optimized HPLC separation of 14 PITC-derivatized amino acids from a standard (top), culture sample (middle) and a standard that had not been derivatized with PITC (bottom).

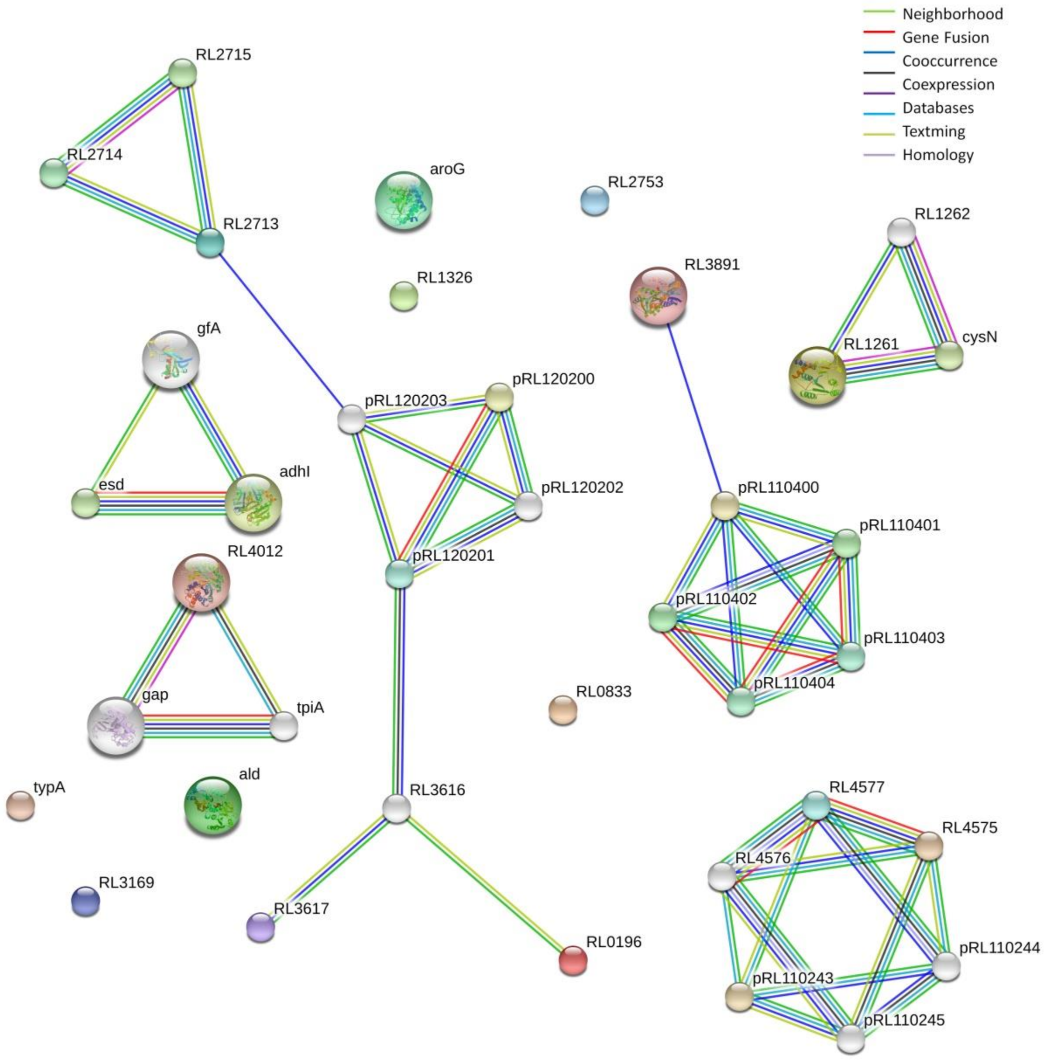

Figure S4. Predicted functional network of differentially expressed proteins constructed using the STRING database. Nodes represent proteins. Lines represent functional relation evidence from fusion (red), neighborhood (green), coocurrence (blue), experiments (purple), text mining (yellow), databases (light blue) and coexpression (black).

Table S1. Calculated molecular weight (mass) and isoelectric point of proteins from each spot isolated by 2D gel electrophoresis unique to either the wild type or *ctpA* mutant. The proteins were focused on 17cm IPG strips (pH 4-7) for a total of 30,000 Vh and followed by SDS PAGE electrophoresis on 10% acrylamide gels.

Wild type strain			*ctpA* mutant strain		
**Spot No.**	**Protein Mass (kDa)**	**Isoelectric point**	**Spot No.**	**Protein Mass (kDa)**	**Isoelectric point**
1	22.05	4.25	1	28.92	4.73
2	19.67	4.16	2	24.16	4.58
3	19.33	4.30	3	23.39	4.68
4	22.84	4.63	4	19.74	4.60
5	29.48	4.61	5	20.29	4.71
6	22.84	4.72	6	18.02	4.69
7	20.25	4.95	7	16.35	4.52
8	22.90	5.14	8	19.76	4.99
9	36.04	5.26	9	20.87	5.25
10	21.06	5.26	10	19.06	5.82
11	19.13	5.43	11	15.68	5.61
12	17.28	5.84	12	15.24	5.76
13	15.65	5.75	13	16.96	5.88
14	26.03	5.81	14	20.50	6.00
15	25.66	6.25	15	20.59	6.30
16	24.46	6.37	16	21.30	6.55
			17	21.55	6.57
			18	28.61	6.64

Table S2. Potential substrates of CtpA in *Rhizobium leguminosarum* bv. *viciae* 3841 identified from 2D gel electrophoresis pI and molecular weight, with isoelectric point and molecular weight before and after cleavage, potential cleavage site and function.

Protein	PI		MW (kDa)		Cleavage site	Function
	**Before**	**After**	**Before**	**After**		
RL0242	5.45	5.29	47.5	45.1	VAL	O antigen biosynthesis protein
RL1750	4.59	4.31	22.5	18.3	VAR	ADP-binding subunit of Dhal, PTS-dependent dihydroxyacetone kinase
pRL110559	9.58	8.93	34.9	31.8	VAT	AraC family transcriptional regulator
RL2341	9.62	9.30	18.86	16.94	AAA	ABC transporter
RL0170	9.38	9.38	30.58	30.27	AAA	Putative ATP-binding component of phosphate uptake ABC transporter
RL3424	6.84	8.37	46.00	44.20	VAA	DctA C4-dicarboxylate transporter
pRL120273	6.30	5.90	46.99	45.00	LAA	DctA C4-dicarboxylate transporter
RL2064	6.48	6.19	66.00	64.5	AAA	Methyltransferase
RL0196	5.40	5.26	34.90	32.14	AAV	Putative outer membrane basic protein, membrane lipoprotein
RL0944	5.35	5.17	20.18	19.61	RAV	Putative LemA-like outer membrane protein
RL1435	9.41	9.56	14.42	13.11	VARGAA	LipA, putative outer membrane protein
RL1635	9.62	9.30	18.86	16.94	SAASAA	Putative outer membrane protein
RL2036	4.79	4.68	45.46	44.37	VAA	Putative outer membrane long-chain fatty acid transport protein precursor
RL3135	6.12	5.71	47.82	44.81	AAL	UgpB, putative glycerol-3-phosphate-binding periplasmic protein for G3P transport
RL3040	5.40	4.83	26.61	24.58	LAA	Putative molybdate-binding periplasmic protein
pRL90111	5.76	5.43	48.81	46.76	AAA	LipA, putative outer membrane protein
pRL90237	5.38	5.36	44.95	42.59	AAL	Putative outer membrane protein
